# Relationship between rest–activity rhythms and cardiorespiratory fitness in middle-aged workers: a cross-sectional study with non-parametric analysis using accelerometers worn on the thigh

**DOI:** 10.1186/s12889-023-17580-w

**Published:** 2024-01-02

**Authors:** Jaehoon Seol, Rina So, Fumiko Murai, Tomoaki Matsuo

**Affiliations:** 1 Research Center for Overwork-Related Disorders, National Institute of Occupational Safety and Health, Japan (JNIOSH), Nagao 6-21-1, Tama-ku, Kawasaki, Kanagawa 214-8582 Japan; 2 International Institute for Integrative Sleep Medicine (WPI-IIIS), University of Tsukuba, Tsukuba, Japan; 3 R&D Center for Tailor-Made QOL, University of Tsukuba, Tsukuba, Japan; 4 Ergonomics Research Group, National Institute of Occupational Safety and Health, Japan (JNIOSH), Kawasaki, Kanagawa Japan

**Keywords:** Cardiorespiratory Fitness, Circadian rhythm, Physical activity, Employee, Occupational Health

## Abstract

**Background:**

Rest–activity rhythms are directly related to health risks, but there are limited objective methods to assess them. This study aimed to investigate the relationship between rest–activity rhythms and cardiorespiratory fitness (CRF) in middle-aged workers.

**Methods:**

Peak oxygen uptake was measured on a treadmill to assess CRF in 254 middle-aged workers who were divided into low, medium, and high-CRF groups based on tertiles. Participants were asked to wear an accelerometer (activPAL) on their thighs for 1 week, and the logarithmically transformed acceleration data were used for the analysis of a 24-hour rest–activity rhythm. Sex, age, body mass index, occupation, smoking status, and alcohol consumption were used as covariates in Model 1, with Model 2 also including walking count on non-workdays. Repeated measures analysis of variance was used to compare time course of rest–activity rhythms changes on workdays between groups, and post-hoc tests were conducted using Bonferroni’s correlation.

**Results:**

Higher CRF correlated with increased physical activity. In model 1, higher CRF showed improved interdaily stability, but the significant difference disappeared in model 2 after adjusting for non-workday walking counts. A time-course group comparison showed that the high group had significantly higher activity levels than those of the low group from 6:00 to 8:59 and 17:00 to 17:59 and the medium group from 6:00 to 7:59 and 19:00 to 19:59.

**Conclusions:**

Workers who have better rest–activity rhythms and engage in higher levels of physical activity on workdays tend to have higher CRF levels. Regular daily routines, influenced by physical activity during holidays, can positively impact cardiopulmonary endurance.

**Supplementary Information:**

The online version contains supplementary material available at 10.1186/s12889-023-17580-w.

## Background

Cardiorespiratory fitness (CRF) is a highly reliable predictor of physical and mental health outcomes and mortality in individuals with or without chronic diseases, including cardiovascular disease and depressive symptoms [1–5].

A 10-year follow-up longitudinal study has revealed that CRF decreases throughout one’s lifespan; however, engaging in sports and/or leisure-time physical activities can help slow down this process [6]. Moreover, increasing physical activity and independently decreasing sedentary behavior are directly associated with higher CRF levels [7]. However, an intervention study that reduced sedentary behavior among middle-aged adults for 1 h per day for 6 months without adding exercise did not affect CRF, while increasing daily activity may increase CRF [8]. Specifically, there is a strong interconnection between the physical activity level and sedentary behavior of workers [9], thereby warranting future studies that focus on occupational cohorts with a substantial prevalence of sedentary behavior at work [9]. Although this may vary according to occupation, workers who spend most of their working hours sitting may have poor CRF [10]. In an epidemiological study using compositional data analysis, reallocation from sedentary behavior to other physical activities (e.g., activities with a low and moderate-to-vigorous intensity) during nonworking time on workdays, including walking to the station, climbing stairs, standing on the train/bus, and engaging in household activities, was favorably associated with cardiometabolic status, which is related to CRF [11], among office workers [12].

Daily circadian synchronization of physiological functioning is essential for the optimal functioning of peripheral organs, including the heart [13]. Prolonged circadian misalignment is associated with increased blood pressure, diminished sleep quality, and an overall increased risk of cardiovascular disease [13]. Specifically, healthy circadian rhythms in adults are independent of physical activity and associated with lower cardiometabolic risk, cardiovascular disease, and mortality [14, 15].

In recent years, accelerometers have become increasingly popular owing to better participant compliance and their ability to measure circadian rhythms, including activity and sleep, over a 24-h protocol [16]. Previous studies with nonparametric analyses used an accelerometer worn on the wrist (e.g., ActiGraph) [17, 18]. These novel approaches have enabled the evaluation of 24-h rest–activity rhythms using accelerometers [19]. However, using wrist movement counts in estimating physical activity (e.g., energy expenditure) may introduce more errors than using other body sites, as there is a significant amount of upper limb gesticulation (e.g., worker’s main activities, such as animated talk, keyboarding, and repetitive desk work) that does not correspond to large body movement [20–23]. Further studies should focus on accelerometers worn in other locations (e.g., activPAL), which measure 24-h rest–activity rhythms [22, 23].

However, only a limited number of studies have investigated the measurement of 24-h rest–activity rhythms using accelerometers worn on the thigh or other locations. In addition, the relationship between 24-h rest–activity rhythms and CRF remains unknown. Based on the results of previous studies [1, 12, 14, 15], we formulated two hypotheses: (1) workers who maintain a regular workday exhibit higher CRF and (2) workers who are more physically active on the workday demonstrate higher CRF.

## Methods

### Participants and data collection

This cross-sectional study was conducted in Japan between June 2016 and March 2022 [24]. All the participants were recruited through a website advertisement. We enrolled participants from Japan’s capital area who were aged between 30 and 59 years old and were employed full-time, meaning they worked for at least 6 h a day and 4 days a week. Additionally, the participants had to have completed a test to measure their CRF (i.e., peak oxygen uptake [VO_2peak_]) and could not take any drugs that affected their autonomic nervous system (e.g., *β*-blockers). The participants also had to have no medical conditions that would preclude them from taking the VO_2peak_ test and had to have received medical certification within the past year. The flow of the day of the visit has been described previously [24]. In total, 320 middle-aged adults participated in this study.

We excluded individuals who were shift workers (n = 24), had unclear occupational status (n = 2), provided less than 4 days of accelerometer data (n = 30), or did not wear the accelerometer during the sleep period (n = 10). Ultimately, 254 participants were included in the analysis.

### Measurements

#### Rest–activity rhythm data acquisition and analysis

The participants were required to continuously wear an accelerometer (activPAL™, Pal Technologies Ltd., Glasgow, UK) for more than 7 consecutive days (24 h/day), except during bathing. The accelerometer data were recorded at 15-s intervals (epochs) over 24 h at a sampling rate of 10 Hz. Over the course of the study, the activPAL model transitioned from activPAL 3 C (2016 to 2017) to activPAL micro (2017 to 2019) and finally to activPAL 4 (2019 ~ 2022). However, all raw data were processed using the same standard algorithm (i.e., VANE) by PAL Software Suite version 8 to extract the acceleration data. The raw accelerometer data (i.e., acceleration volume) were computed using PAL Software Suite version 8 (Pal Technologies Ltd., Glasgow, UK) [25, 26]. In addition, study participants were instructed to use a web diary (web application) developed by us to record their bedtimes, wake times on workdays and off-days, the start of work, return home, and exercise. The weekly accelerometer data were exclusively computed based on the data collected during workdays with reference to the web diary. The calculated acceleration data were log-transformed as in previous studies [19] and represented over a 24-h time period (Additional file 1: Figure S1). The *GGIR* R package (version 2.10.1) was used to characterize and impute missing acceleration data [27]. Data selection and exclusion criteria were based on a previous study [17].

A nonparametric methodological analysis of rest–activity rhythm measures was derived using the *nparACT* R package (version 0.8) [28], including variables such as interdaily stability (IS), intradaily variability (IV), and relative amplitude (RA). We also calculated the amplitude of acceleration during the 5 h with the lowest activity (L5) and the 10 h with the highest activity (M10), to determine the extent of activity during the rest and active periods [19].

**Fig. 1:**
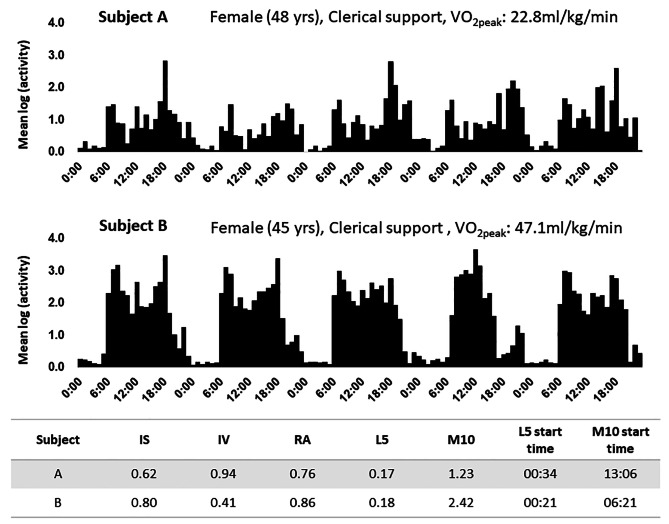
Examples of different rest and activity rhythms among female office workers of the same age. Bars indicate hourly data for the workday. IS, interdaily stability; IV, intradaily variability; RA, relative amplitude; L5, amplitude of acceleration during the 5 h with the lowest activity; M10, amplitude of acceleration during the 10 h with the highest activity

IS describes the invariability of the 24-h rhythm between different days and indicates better coupling/synchronization of the rest–activity rhythm to external zeitgebers. Conversely, IV measures the degree of fragmentation in a rhythm, including the frequency and extent of transitions that may reflect the occurrence of daytime naps and/or nocturnal awakenings. A perfect sine wave would have an IV value close to 0, whereas Gaussian noise would have a value of approximately 2, and a rhythm with a well-defined ultradian period of approximately 2 h could have an even higher value. RA describes the normalized difference between M10 and L5, with higher values indicating a healthy 24-h rhythm, reflecting higher activity during the wake period and relatively lower activity at night [19]. Briefly, a healthy rhythm is characterized by greater activity during active periods and lower activity during rest periods. These indices have been extensively used in the study of rest–activity rhythms and are valuable tools for quantifying the properties of biological rhythms [17, 19, 28] (Fig. 1).

#### Cardiorespiratory fitness

The participants underwent a graded exercise test on a treadmill (AR200; Minato Medical Science, Osaka, Japan) following the Bruce protocol to directly measure their VO_2peak_. Throughout the test, an open-circuit computerized indirect calorimeter (AE-310 S, Minato Medical Science, Osaka, Japan) was used to continuously measure ventilation and expired gases, whereas heart rate was monitored using an electrocardiogram (Life Scope, Nihon Kohden, Tokyo, Japan). Moreover, the rate of perceived exertion was recorded using the Borg 6–20 scale.

VO_2peak_ was determined as the highest 30-s average VO_2_ value that met at least three of the following four criteria: (1) a respiratory exchange ratio exceeding 1.10; (2) a maximal heart rate within 10 beats per minute of the age-predicted maximum; (3) a rate of perceived exertion exceeding 17; and (4) a plateau in VO_2_ despite further increases in workload [24, 29]. The measurement methods and other details are explained in a previous study [24].

#### Potential confounders

As potential confounding factors, we included age (continuous variable), sex (male or female), body mass index (BMI) (continuous variable), alcohol consumption (never, 1–2 times/month, 1–2 times/week, 3–4 times/week, 5–6 times/week, and daily), smoking history (past/never or current), walking count on non-workdays measured as active Pal, and occupational information.

The occupation data were classified based on the Japanese Standard Occupational Classification [30] into various categories, including managers, clerical workers, marketing professionals, service and sales personnel, manual laborers, healthcare professionals, education professionals, technicians and researchers, security personnel, agricultural/forestry and fishery workers, manufacturing personnel, transport and machinery workers, construction and mining workers, and others. We reclassified the occupation data into five categories: (1) clerical support; (2) services and sales; (3) professionals; (4) skilled agricultural, forestry, and fishery workers and plant/machine operators and assemblers; and (5) others.

### Statistical analyses

To remove the influence of sex and age [31, 32], we stratified the patients into 5-year age increments (30–34, 35–39, 40–44, 45–49, 50–54, and 55–59 years old). Subsequently, we defined tertiles of VO_2peak_ into the low (32.6 ± 4.4 ml/kg/min, n = 80), moderate (38.2 ± 4.4 ml/kg/min, n = 91), and high groups (44.9 ± 5.5 ml/kg/min, n = 83) (Table 1).

**Table 1:** Sex and age stratification for peak oxygen uptake

	Age category	1st tertile: Low group	2nd tertile: Middle group	3rd tertile: High group
Female, n	30–34 y	2 (29.4–30.2)	3 (32.8–43.2)	2 (43.7–44.8)
	35–39 y	4 (26.3–33.2)	5 (33.7–35.7)	4 (38.5–43.8)
	40–44 y	7 (27.8–33.6)	7 (33.7–38.4)	7 (38.6–51.8)
	45–49 y	10 (29.4–30.2)	11 (32.1–36.3)	10 (36.4–47.5)
	50–54 y	8 (22.2–29.6)	9 (30.0–34.0)	8 (34.9–44.4)
	55–60 y	5 (26.8–32.2)	6 (33.4–36.0)	5 (37.2–41.8)
Male, n	30–34 y	3 (39.3–43.9)	4 (44.7–48.1)	4 (49.5–58.2)
	35–39 y	4 (31.9–41.8)	5 (42.7–46.2)	5 (48.0–50.7)
	40–44 y	7 (33.6–39.4)	8 (39.9–44.1)	7 (44.3–56.4)
	45–49 y	12 (26.2–38.8)	13 (39.7–45.1)	12 (45.5–54.6)
	50–54 y	10 (31.9–37.7)	11 (37.8–41.0)	11 (42.0–51.6)
	55–60 y	8 (30.3–36.2)	9 (36.4–41.7)	8 (42.0–48.3)
Total, n		80	91	83

Note: Parentheses indicate the minimum and maximum values of VO_2peak_ (ml/kg/min). VO_2peak_ was stratified by sex and age into tertiles: low (32.6 ± 4.4 ml/kg/min, n = 80), moderate (38.2 ± 4.4 ml/kg/min, n = 91), and high groups (44.9 ± 5.5 ml/kg/min, n = 83)

To compare the three groups as independent variables with participant characteristics as dependent variables, we used a one-way analysis of variance with Bonferroni post-hoc tests for continuous variables and the chi-squared test for categorical variables. An analysis of covariance (ANCOVA) trend test was conducted to examine the trend of rest–activity rhythm variables as dependent variables, with the three groups as independent variables. To ensure accurate analysis and account for potential confounding factors related to non-workday physical activity, three models were utilized: an unadjusted model, Model 1 (adjusted for age, sex, BMI, alcohol consumption, smoking history, and occupational information), and Model 2 (which included all the factors in Model 1 and walking count on non-workdays). In this study, time-course changes were analyzed with groups as independent variables and rest–activity rhythms as dependent variables over a 24-hour period, using repeated-measures ANCOVA while controlling for age, sex, BMI, alcohol consumption, smoking history, and occupation information. The Bonferroni correction was performed as a post-hoc test. For this, the acceleration data for rest–activity rhythms were calculated in 15-s epoch increments and reported as an hourly average. All statistical analyses were performed using the Statistical Package for the Social Sciences version 26.0 (IBM Corporation, Armonk, NY, USA). Statistical significance was set at *P* < 0.05 (two-tailed).

## Results

The average number of days with valid accelerometer data was 6.2 ± 0.9 days. The participants’ average VO_2peak_ was 38.6 ± 6.9 ml/kg/min, and 44.5% of the total participants were female. Table 2 shows the notable variations in BMI, VO_2peak_, and walking count on workdays and non-workdays among the CRF groups. However, no significant differences were observed in occupational status among the CRF groups.

**Table 2:** Characteristics of participants

Variables	Low group (n = 80)	Middle group (n = 91)	High group (n = 83)	*P* for Trends	ANOVA or chi square *P* value
	Mean ± SD	Mean ± SD	Mean ± SD		
Age, years	47.1 ± 7.3	46.6 ± 7.4	46.7 ± 7.3	0.736	0.904
Male, n (%)	44 (55.0)	50 (54.9)	47 (56.6)	-	0.969
BMI, kg/m^2^	24.6 ± 3.5	22.5 ± 3.0^#^	21.8 ± 2.2^#^	< 0.001	< 0.001
Smoking history, n (%)	48 (60.0)	65 (71.4)	58 (69.9)	-	0.235
Alcohol consumption (More than 1 time/month), n (%)	57 (71.3)	60 (66.7)	62 (74.7)	-	0.507
VO2_peak_, ml/kg/min	32.6 ± 4.4	38.2 ± 4.4^#^	44.9 ± 5.5^#*^	< 0.001	< 0.001
Walking count on workdays, count	9917 ± 3289	11,210 ± 3820	11,904 ± 4543^#^	< 0.001	0.005
Walking count on non-workdays, count	9063 ± 5523	9916 ± 4649	11,038 ± 4801^#^	< 0.001	0.044
**Occupation categories**					
Clerical Support, n (%)	35 (43.8)	45 (49.5)	33 (39.8)	-	0.432
Services and sales, n (%)	22 (27.5)	22 (24.2)	22 (26.5)	-	0.877
Professionals, n (%)	18 (22.5)	19 (20.9)	20 (22.4)	-	0.879
Skilled agricultural, forestry, fishery workers, and plant/machine operators and assemblers, n (%)	5 (3.8)	3 (3.3)	4 (3.9)	-	0.871
Others, n (%)	2 (2.5)	2 (2.2)	4 (4.8)	-	0.566

Note: BMI, body mass index; ANOVA, analysis of variance; SD, standard deviation; VO_2peak,_ peak oxygen uptake; ^#^significant difference compared to low group; ^*^Significant difference compared to middle group; VO_2peak_ was stratified by sex and age into tertiles: low (32.6 ± 4.4 ml/kg/min, n = 80), moderate (38.2 ± 4.4 ml/kg/min, n = 91), and high groups (44.9 ± 5.5 ml/kg/min, n = 83)

In all models, higher CRF was associated with higher M10 values. Conversely, IS values tended to show better outcomes with higher CRF, but the significant difference disappeared in model 2, which accounted for walking counts on non-workdays. Similarly, the unadjusted model indicated that higher cardiopulmonary endurance was associated with better RA values, but the significant difference disappeared in models 1 and 2 (Table 3).

**Table 3:** Comparison of rest–activity rhythm variables between individual groups

Variables	Low group (n = 80)		Middle group (n = 91)		High group (n = 83)		*P* for Trends
	Mean	(SE)	Mean	(SE)	Mean	(SE)	
Interdaily stability, index							
Unadjusted	0.654	(0.016)	0.669	(0.015)	0.699	(0.016)	0.052
Model 1	0.650	(0.017)	0.670	(0.015)	0.702	(0.016)	0.042
Model 2	0.655	(0.017)	0.670	(0.015)	0.694	(0.017)	0.126
Intradaily variability, index							
Unadjusted	0.771	(0.024)	0.787	(0.022)	0.718	(0.023)	0.116
Model 1	0.777	(0.025)	0.787	(0.022)	0.712	(0.024)	0.071
Model 2	0.774	(0.025)	0.780	(0.022)	0.720	(0.024)	0.141
Relative amplitude, index							
Unadjusted	0.769	(0.012)	0.797	(0.011)	0.804	(0.012)	0.042
Model 1	0.771	(0.013)	0.795	(0.011)	0.803	(0.012)	0.093
Model 2	0.773	(0.013)	0.797	(0.012)	0.801	(0.013)	0.134
L5, log-transformed activity							
Unadjusted	0.266	(0.019)	0.229	(0.017)	0.240	(0.018)	0.325
Model 1	0.264	(0.020)	0.231	(0.018)	0.241	(0.019)	0.431
Model 2	0.263	(0.020)	0.231	(0.018)	0.241	(0.019)	0.456
M10, log-transformed activity							
Unadjusted	1.981	(0.053)	1.973	(0.050)	2.172	(0.052)	0.011
Model 1	1.964	(0.057)	1.974	(0.051)	2.187	(0.055)	0.010
Model 2	1.962	(0.056)	1.989	(0.050)	2.173	(0.054)	0.015
L5 start time, hour							
Unadjusted	25.059	(0.144)	25.077	(0.135)	24.777	(0.141)	0.164
Model 1	25.047	(0.153)	25.089	(0.136)	24.787	(0.146)	0.240
Model 2	25.041	(0.155)	25.073	(0.138)	24.843	(0.150)	0.381
M10 start time, hour							
Unadjusted	10.113	(0.334)	9.843	(0.314)	9.700	(0.328)	0.379
Model 1	10.066	(0.352)	9.893	(0.313)	9.729	(0.335)	0.507
Model 2	10.086	(0.354)	9.872	(0.315)	9.811	(0.341)	0.593

Note: Model 1 was adjusted for age, sex, BMI, alcohol consumption, smoking history, and occupation information; Model 2 included all the factors in Model 1, plus walking step count on non-workdays; SE, standard error; L5, amplitude of acceleration during the 5 h with the lowest activity; M10, amplitude of acceleration during the 10 h with the highest activity; BMI, body mass index; VO2_peak_ was stratified by sex and age into tertiles: low (32.6 ± 4.4 ml/kg/min, n = 80), moderate (38.2 ± 4.4 ml/kg/min, n = 91), and high groups (44.9 ± 5.5 ml/kg/min, n = 83)

**Fig. 2:**
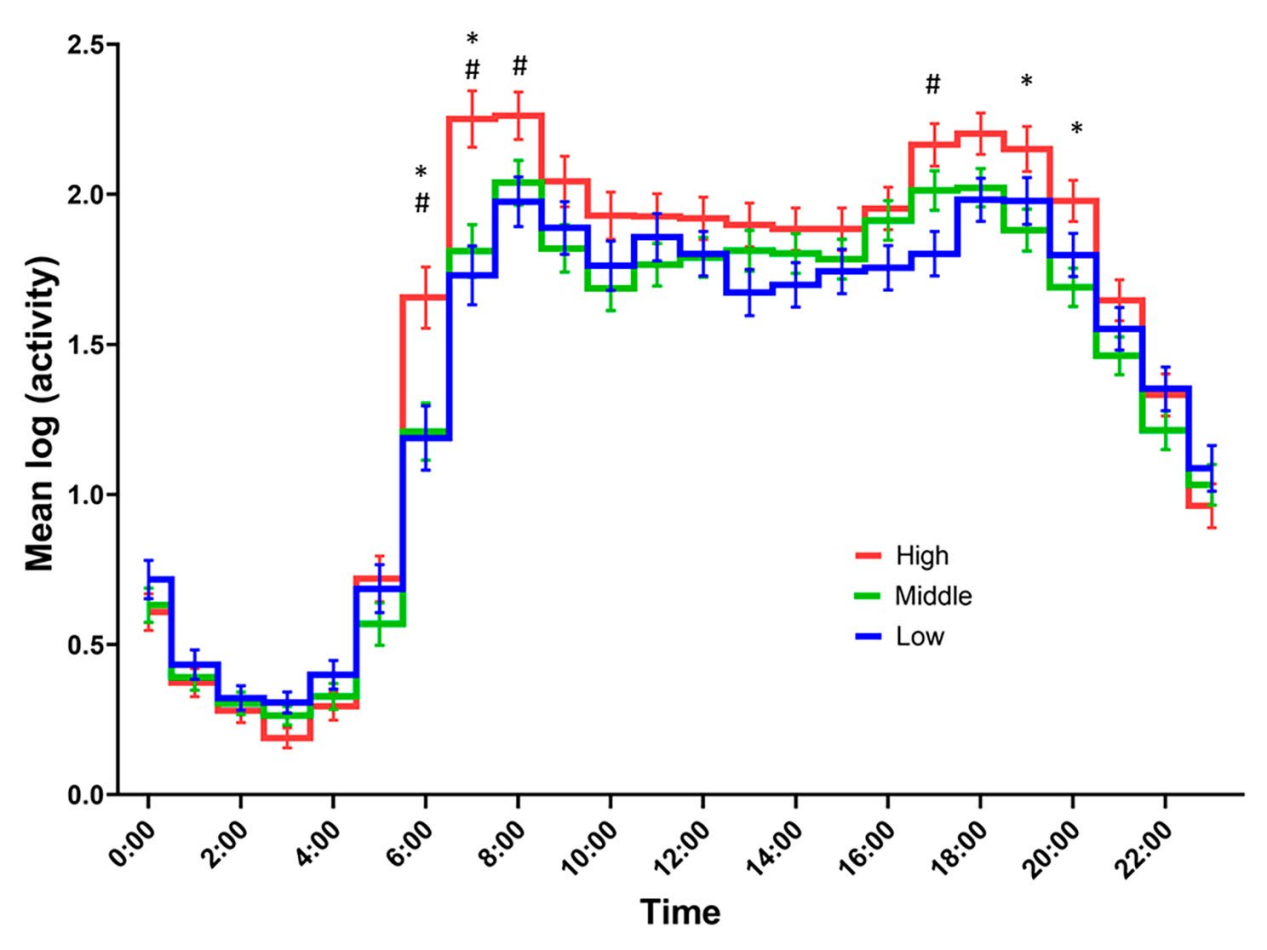
Time course of 24 h rest–activity rhythm during workdays. The time course of 24 h rest–activity rhythm during workdays indicated an average of 1 h using two-way analysis of covariance; ^#^Significant difference between the low-CRF group and the high-CRF group; ^*^Significant difference between the medium-CRF group and the high-CRF group; VO_2peak_ was stratified by sex and age into tertiles: low (32.6 ± 4.4 ml/kg/min, n = 80), moderate (38.2 ± 4.4 ml/kg/min, n = 91), and high groups (44.9 ± 5.5 ml/kg/min, n = 83). CRF, cardiorespiratory fitness; VO_2peak_, peak oxygen uptake

The rest–activity data over time showed significant effects of time (*P* < 0.001), group (*P* = 0.016), and interaction (*P* < 0.001). The post-hoc test results showed that the high-CRF group was significantly more active at 6:00–8:59 and 17:00–17:59 (all *P* < 0.05) compared with that of the low-CRF group (Fig. 2). In addition, the high-CRF group was significantly more active at 6:00–7:59 and 19:00–20:59 compared with that of the medium-CRF group (all *P* < 0.05) (Fig. 2). However, no significant differences were observed during nighttime (21:00–5:59) and working time (9:00–16:59) among the groups (Fig. 2).

## Discussion

This study examined the relationship between rest–activity rhythms and CRF levels in middle-aged workers. Rest–activity rhythms were measured using an accelerometer worn on the thigh. The participants with higher levels of CRF were more active, regardless of whether it was a workday or not, compared with those with lower levels of CRF (Table 2).

The present study results showed that workers with higher CRF tended to have more regular workdays, supporting our first hypothesis (IS in Model 1, Table 3). However, the amount of exercise on non-workdays may also play a role (IS in Model 2, Table 3), suggesting that individuals with irregular lifestyle patterns can offset their impact on CRF through weekend exercise [33].

Furthermore, the results support the second hypothesis, indicating that workers with higher weekday physical activity may maintain elevated CRF levels regardless of the amount of physical activity outside of the weekday (M10 in Table 3). Specifically, participants with higher levels of CRF exhibited higher M10 values and were notably more active during their commute to work and during equivalent leisure time after work compared to those of workers with low or medium CRF (Table 3; Fig. 2).

The present study is one of the few studies to investigate the 24-hour rhythm of individuals with high CRF who are engaged in the workforce, using accelerometers to objectively assess their rest–activity rhythm, with a focus on workdays. The findings from this study partly align with previous research results, which has shown a favorable association between physical activity during non-work hours on weekdays and cardiometabolic status related to CRF and mortality [11–15].

A previous observational study demonstrated that sleep duration was negatively associated with CRF in younger adults [34]. However, the sleep-related variable L5 (similar to wake after sleep onset) was not significantly related to CRF. This can be attributed to the fact that the sleep patterns examined in this study are specifically limited to workdays; therefore, the evaluation of sleep duration is not based on individual preference or choice. CRF is a highly reliable predictor of cardiovascular disease [1–5, 11–15]. Additionally, engaging in both vigorous and light physical activities toward the end of a workday is positively associated with enhancing employees’ self-efficacy while working, which in turn benefits their work roles [35].

Relative workload (e.g., heart rate reserve) determines the acute and long-term physiological effects of physical activity at work. Increasing CRF decreases the relative workload and reduces the risk of cardiovascular disease and all-cause mortality, as reported in a previous study [36]. Hence, the physical activity level of workers may affect their physical and mental health, which may in turn affect their CRFs. Another concept is the physical activity paradox, which suggests that higher levels of physical activity during work increase the risk of cardiovascular disease [36–38]. However, our results contradicted this paradox, possibly because previous studies primarily focused on blue-collar workers, whereas our study largely involved white-collar workers (93.8%). In Japan, there is a phenomenon known as overwork-related sudden death, or “*karoshi*,” which is associated with cerebrovascular, cardiac, and psychiatric conditions. Cardiovascular and cerebrovascular diseases are highly interconnected, and comorbidities or complications from both diseases can result in high mortality rates [39]. The findings of this study suggest that engaging in physical activity during non-working hours on workdays may protect against cardiovascular disease.

Some previous studies have revealed a relatively high agreement between ActiGraph and activPAL recordings in free-living conditions [40–42], although there are also some arguments that activPAL may be slightly less sensitive than ActiGraph; moreover, activPAL may underestimate or overestimate sleep duration compared with that using ActiGraph [43, 44]. This disagreement is based on the cutoff point for determining moderate-to-vigorous physical activity and the appropriate algorithm for measuring sleep using accelerometers [45]. A systematic review has stated that it is essential to use accelerometers worn at various sites for 24 h to accurately measure rest–activity rhythms [22, 23]. Our results, which calculated rest–activity rhythms on a timeline (Additional file 1: Figure S1), appear to be similar to those of a previous study [19]; however, future studies should confirm the agreement between ActiGraph and ActivPAL rest–activity rhythm measurements.

Our study has some limitations. First, the sample size was small. Thus, although our analysis was adjusted for occupational status and other potential confounders, future studies should focus on individual differences, including sex, age, and occupational status. Second, we used prior studies [31, 32] to stratify participants to minimize the effects of sex and age; however, caution should be exercised in generalizing our results because of potential sampling bias in the selection of participants. Finally, owing to the limited duration of measurement (i.e., consecutive 7 days), the investigation of rest–activity rhythms during non-workdays was not feasible [23]. We adjusted the number of steps to account for the influence of non-workday exercise habits; however, there was still a positive correlation between the number of steps taken on non-workdays and workdays (*r* = 0.345, *P* < 0.001), which raises the possibility of over-adjustment. Thus, future studies should aim to conduct measurements over a period of > 2 weeks to accurately assess rest–activity rhythms during non-workdays.

## Conclusions

Participants who have healthy daily rest–activity rhythms and are more physically active during working hours have higher levels of CRF. Notably, the amount of physical activity while attending and leaving work on workdays and leisure time after work may also have significant effects on the CRF levels of working individuals.

### Electronic supplementary material

Below is the link to the electronic supplementary material.

Additional file 1: Figure S1. Rest and activity pattern of the entire sample (n = 254) aggregated to a single 24-h period

## Data Availability

The datasets used and/or analyzed during the current study are available from the corresponding author (seol.jaehoon.ge@u.tsukuba.ac.jp) on reasonable request.

## Abbreviations

CRF: Cardiorespiratory fitness
VO_2peak_: Peak oxygen uptake
ANCOVA: Analysis of covariance
BMI: Body mass index
IS: Interdaily stability
IV: Intradaily variability
L5: Amplitude of acceleration during the 5 h with the lowest activity
M10: Amplitude of acceleration during the 10 h with the highest activity
