# Supplementary material for: Relationship between rest–activity rhythms and cardiorespiratory fitness in middle-aged workers: a cross-sectional study with non-parametric analysis using accelerometers worn on the thigh
Source: BMC Public Health. 2024 Jan 2;24:62. doi: 10.1186/s12889-023-17580-w (PMC10763488; doi:10.1186/s12889-023-17580-w)
Supplement: Supplementary file 1 — Additional file 1: Figure S1. Rest and activity pattern of the entire sample (n = 254) aggregated to a single 24-h period [file 12889_2023_17580_MOESM1_ESM.docx]

**Figure S1. Rest and activity pattern of the entire sample (n = 254) aggregated to a single 24-h period**

**
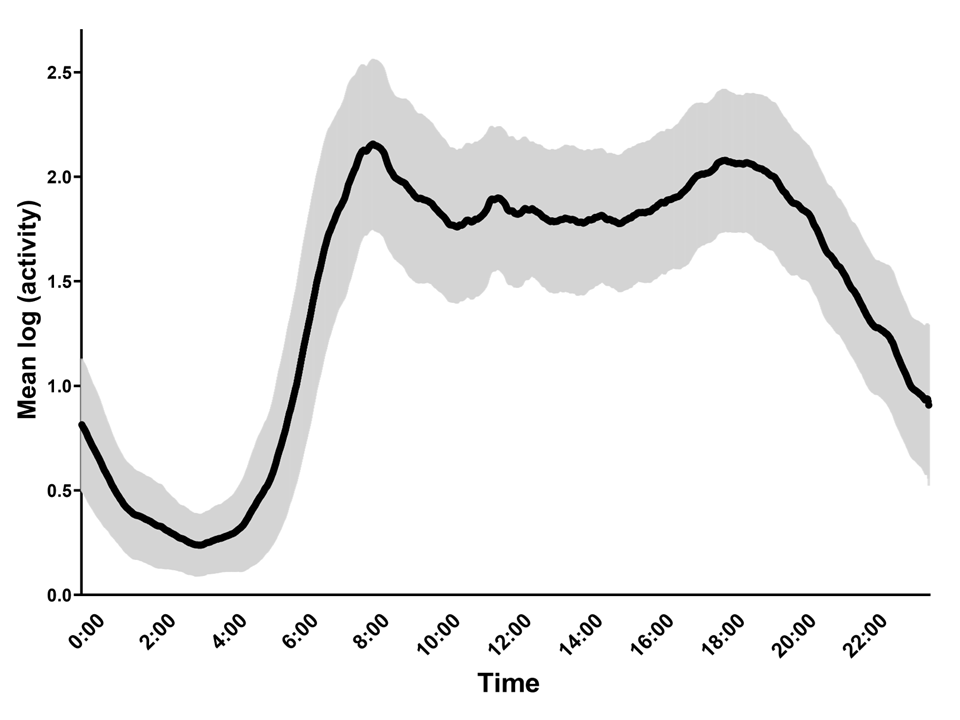
**

The gray ribbon represents +/– 0.5 standard deviation.
